# Extracervical Approaches to Thyroid Surgery: Evolution and Review

**DOI:** 10.1155/2019/5961690

**Published:** 2019-08-20

**Authors:** B. M. Sephton

**Affiliations:** Imperial College Healthcare NHS trust, London, UK

## Abstract

Over the last two decades, advances and adaptation of technology have led to a variety of endoscopic thyroidectomy procedures being performed. The drive for extracervical procedures has been predominantly influenced by the desire for improved cosmesis via avoidance of visible scars. Extracervical techniques have shown considerable evolution with approaches that have included transaxillary, breast, postauricular, and transoral routes. There has been a varied evidence base for each of these approaches with regard to technical feasibility, safety, patient satisfaction, and cost-effectiveness. In recent years, robotic-assisted thyroid surgery has gained increased popularity worldwide with the introduction of the da Vinci Robot. Reports of improved postoperative outcomes and patient satisfaction have been in contrast to the financial burden, longer operative time, and increased training required which, to date, have limited widespread application. The aim of this review is to describe the evolution of extracervical procedures including surgical approaches, outcomes, advantages, and disadvantages. Consideration is also given to the future direction of extracervical thyroid surgery with regard to the safety, feasibility, and application of robotic systems.

## 1. Introduction

Thyroid surgery has progressed considerably over the past 30 years from the original surgical approach of an open thyroidectomy performed through an 8-10 cm collar incision [1]. Currently, open thyroidectomies are typically performed through a 4-6 cm transverse incision made in the anterior lower neck. Towards the end of the twentieth century, new techniques for thyroidectomy were developed to include both minimally invasive and extracervical remote access surgery. Minimally invasive surgical techniques have attracted interest since the 1980s because these procedures enable the level of “physical invasiveness” and the size of the skin incision to be reduced [2, 3].

In 1996, Gagner described the first endoscopic subtotal parathyroidectomy for hyperparathyroidism obtaining good clinical and cosmetic results [4]. The technique progressed to include total thyroidectomy and subsequent studies reported no major complications, better cosmetic results, and an earlier return to activity when compared to conventional thyroidectomy [5, 6]. Technical modifications to the original minimally invasive thyroidectomy (MIT) techniques have been continually tried to further improve the results of MIT and such experimentation led to the development of minimally invasive video-assisted thyroidectomy (MIVAT). The most well researched MIVAT technique is that developed by Paolo Miccoli et al. in 1998 [7] and is the endoscopic method that has become most widespread to date. The MIVAT technique uses a 1.5 cm skin crease incision above the sternal notch. Due to the smaller neck incision and decreased dissection, MIVAT is associated with improved patient satisfaction, less postoperative pain, decreased length of stay, and less surgical complications than open thyroidectomy [1, 8].

The direct endoscopic approach is considered the least invasive of the MIT procedures. Despite this, the direct approach still leaves incision scars within a highly visible area of the neck. These scars are often well tolerated; however, in certain individuals, the scar may still be problematic and lead to a heightened sense of self-consciousness [9]. Extracervical thyroidectomy removes the incision from the neck to the chest, breast, axilla, or postauricular areas but it is not truly minimally invasive surgery because of the additional surgical dissection required from the remote site. These indirect techniques may, therefore, be classified as a minimal access but maximally invasive approach (MAMIA) [10]. The techniques are classified in terms of where the surgical trocars are introduced and the site of approach has an intimate relationship with cosmetic outcome, safety, and level of invasiveness. The approaches currently utilised are the axillary approach [11, 12], the anterior/breast approach [13, 14], the axillary-bilateral breast approach [15], the bilateral axillo-breast approach [16], the postauricular approach [17], and the transoral approach [18].

The preponderance of females among patients requiring thyroid surgery, particularly in adolescents where cosmesis is of great importance, has been an influence in the development of extracervical approaches [19]. Farahati et al. [20] found that the F:M ratio of thyroid disease can reach as high as 14:1 in some study populations, with the difference in incidence peaking during puberty. Cultural influences have also played a significant part; many of the techniques have been developed in Asia where visible scarring is socially stigmatized [1]. Extracervical techniques have evidence to show technical feasibility with good cosmetic results [15–17, 21–24]; however, to date, these approaches have not been widely accepted with many studies carried out in specialist centers in the hands of experienced thyroid surgeons. This may be due to the more technically demanding nature of the procedure requiring extensive subcutaneous dissection [10].

In recent years, application of robotic techniques has led to advances in extracervical thyroid surgery [25]. The use of robotics can overcome some of the limitations of extracervical approaches; advantages include a three-dimensional view of the operating field, a greater degree of movement from the use of ‘wristed' instruments, and the elimination of hand tremors [24, 26–28]. The lack of working space and involvement of critical nerves and vessels initially delayed application of robotic extracervical approaches; however, in 2009, a South Korean team published the first large series (n=100) of robotic-assisted endoscopic thyroidectomy via an axillary approach using the ‘da Vinci' robotic surgical system (Intuitive Surgical, USA) [29]. Since then, a range of different approaches have been described: transaxillary, retroauricular (also known as the ‘facelift' approach), and trans-oral. The transaxillary and retroauricular approaches are the most common approaches and the most well described in the literature. Initial enthusiasm led to widespread uptake of robotic extracervical thyroid surgery in the Far East [30–32]; however, in the Western World, uptake has been much slower [33].

**Figure 1:**
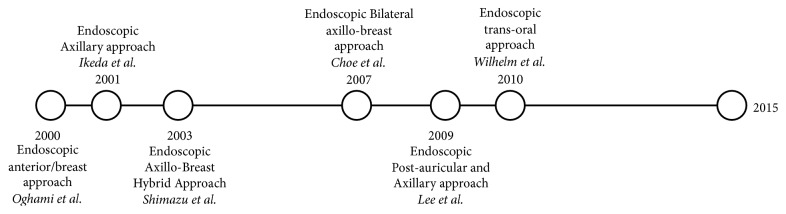
Evolution of endoscopic approaches.

**Figure 2:**
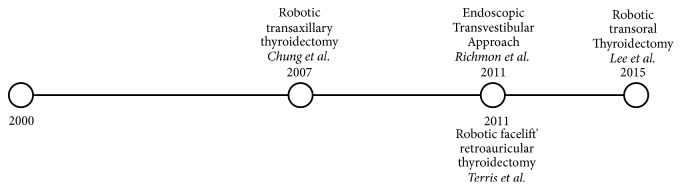
Evolution of robotic approaches.

The aim of this narrative review is firstly to discuss evolution of endoscopic extracervical approaches (Figure 1) including surgical technique, outcomes, and complications. Secondly, the review discusses the development of robotics on extracervical thyroid surgery (Figure 2) leading ultimately to the question of whether or not these approaches are a feasible option to be incorporated into widespread surgical practice for thyroid pathology.

## 2. Endoscopic Anterior/Breast Approach

### 2.1. Surgical Pathway

In 2000, Ohgami et al. [34] developed the anterior/breast approach for performing endoscopic thyroidectomy. The surgical pathway and procedure are fully described by Ohgami in a paper presenting the results of the anterior breast approach on a series of 5 patients [34]. The patient is placed in the supine position on the operating table with the neck extended to expose the surgical area. A 15mm transverse skin incision is made at the parasternal border of the breast. The subcutaneous tissues of the anterior chest and the subplatysmal space can then be dissected bluntly using a dissector. A 12 mm trocar is inserted through the transverse skin incision. The working space is maintained using CO_2_ gas insufflation at a pressure of 5-6 mmHg. At the upper margin of both circumareolar areas, a trocar is inserted: one 10 mm and one 5 mm port.

Further blunt dissection using an ultrasonically activated scalpel ensures an adequate working space above the infrahyoid (strap) muscles is created. The thyroid gland, on the side of the lesion, is completely exposed following dissection of the strap muscles. The procedure is then similar to that of open thyroidectomy, and initial dissection of the gland is started inferiorly at the lower pole and then moves posterolaterally, achieving good elevation and exposure of the gland. Dissection of the thyroid gland and division of the thyroid vessels and parenchyma are achieved with use of an ultrasonically activated scalpel with recognition and preservation of the RLN. The superior thyroid artery and vein are divided last and the specimen is retrieved via the parasternal port.

### 2.2. Indications

Since its introduction in 2001, endoscopic thyroidectomy via the anterior/breast approach has been consistently performed on patients with benign thyroid nodules <5 cm and follicular neoplasm confirmed by preoperative fine-needle aspiration cytology [23, 35]. Some experts now perform this technique for well-differentiated thyroid carcinoma and Graves' disease [36], although this should be limited to 100 g goiters [13]. Current exclusion criteria for the anterior/breast approach include previous open neck surgery, thyroiditis diagnosed by preoperative biochemistry or ultrasound (US), a history of breast malignancy, and substernal goitres. Caution must be taken when considering this surgery for men, due to smaller amounts of subcutaneous fat in the region, and those with well-differentiated tumours over 10 mm in diameter [35].

### 2.3. Surgical Outcomes

In the initial research by Ohgami et al. [34], 5 patients were treated successfully via the anterior/breast approach. All patients underwent a successful hemithyroidectomy, no conversions to an open approach were required, and there were no postoperative complications. The endoscopic view was described as excellent and the RLN and parathyroid glands were identified in all individuals. Only minimal subcutaneous emphysema around the neck was observed with no hypercapnic complications with CO_2_ insufflation methods. Mean operative time was 226 min (range 177-281 min). The resultant scars were all covered by the patient's underwear and all patients were satisfied with the cosmetic result. A later study in 2008 by Sasaki et al. [37] published the results of 92 patients with benign thyroid disease who underwent thyroidectomy via the anterior/breast approach in one institution. This study included a cosmetic satisfaction score, which ranged from 0 to 10: extremely dissatisfied to extremely satisfied. The overall mean satisfaction score was 9.3 with recorded scores highest in the youngest population studied (20-29 years). A later study by Tan et al. [38] in 2015 compared 34 patients undergoing thyroidectomy via the breast approach to 30 patients undergoing conventional open thyroidectomy. All patients had papillary thyroid carcinoma with a diameter of less than 2cm and underwent prophylactic ipsilateral central compartment node dissection as a result. Outcomes were not statistically significant between groups in terms of number of dissected lymph nodes, number of positive lymph nodes, and presence of lymph node metastases suggestive that ipsilateral central lymph node dissection can be safely performed via the anterior breast approach.

In 2003, Park et al. [23] reported the results of 100 patients undergoing the anterior/breast approach and concluded that the anterior/breast approach is a feasible and safe procedure for resection of thyroid nodules provided that a low CO_2_ insufflation pressure is used. A potential concern with endoscopic surgery in the neck is CO_2_ insufflation complications. Gagner [4] and Gottlieb et al. [39] reported severe subcutaneous emphysema and hypercarbia when performing initial parathyroid endoscopic surgeries using CO_2_ insufflation. In these reports, however, a relatively high pressure level of CO_2_ insufflation (15-20 mmHg) was applied. In extracervical approaches, since only the platysma muscle needs to be lifted, lower insufflation pressures of < 4 mmHg are sufficient. No adverse effects have been recorded when such low insufflation pressures are used throughout the different approaches, including both the axillary and anterior/breast approaches [40–42].

In the study by Ohgami [34], initial procedures had a long average operation time, recorded at 226 min; however, a learning curve is to be expected when a novel technique is introduced. In the study by Park et al. [23], the average operation time over the first 2 years was 136 min; however, this was reduced to 67 min in the third year. In 2009, Liu et al. [43] proposed that a learning curve of around 150 operations is needed before a surgeon has an advanced level of skill, proficiency, and stability with endoscopic thyroidectomy. Liu et al. [43] therefore suggest that it is difficult to compare such novel techniques, until a certain level of experience has been reached.

In 2010, Zhang et al. [44] evaluated the surgical invasiveness of the anterior/breast approach. The study population included 24 individuals undergoing the anterior/breast approach and 19 who underwent conventional thyroid surgery. The operation time was not significantly different between the two groups. The endoscopic group had a reduced length of incision and a lower volume of blood loss. Complications such as hypercapnia, RLN palsy, and hypocalcaemia were not observed in either study group. Subjective postoperative pain was recorded using a visual analogue scale (VAS). According to the results from the VAS, the reported severity of pain on the first day was significantly higher in the conventional group than the endoscopic group; however, there was no statistical difference found 48 hours after surgery. Objective postoperative markers of invasiveness were recorded using inflammatory markers (interleukin-6 (IL-6) and C-reactive protein (CRP)). The inflammatory markers were significantly higher in the conventional surgery group compared to the endoscopic group after both 24 and 48 hrs.

To date, widespread application of the anterior/breast approach has been limited. The reasons cited for this are similar: longer operating times, significantly, when the surgeon is not fully proficient [43], the need for additional surgical equipment, and the need for surgical training. These disadvantages led to continued development of further approaches including the axillo-breast (hybrid) approach, a modification of this original technique.

## 3. Endoscopic Axillary Approach

### 3.1. Surgical Pathway

Ikeda et al. [21] presented initial results of axillary approach thyroid surgery in 2001 and demonstrated that it could be performed successfully with a minimal complication rate. The surgical pathway for the technique has been well described, both in Ikeda's original article and subsequent texts [10, 21, 45, 46]. The arm on the operative side is raised exposing the axilla. The incision is placed in the axilla so that the natural resting position of the arm will conceal the scar. Two 5 mm trocars are inserted through a 30 mm skin incision; CO_2_ insufflation occurs at a pressure of 4 mmHg and a flexible laparoscope is inserted. Another 5 mm trocar is then placed near the skin incision. The subplatysmal space is then enlarged via blunt dissection with division of the strap muscles, using an ultrasonically activated scalpel, to expose the ipsilateral lobe of the thyroid gland. Vessels are then identified and ligated using an ultrasonically activated scalpel. Next the gland is retracted medially. The recurrent laryngeal nerve (RLN) is identified and separated carefully from the thyroid gland followed by ligation of the inferior thyroid artery. To completely release the thyroid gland from the trachea, Berry's ligament must be dissected using endoscopic scissors and a dissector. The isthmus of the thyroid is divided using the ultrasonic device and the thyroid gland can be freed. The specimen is extracted through the 30 mm skin incision. The wound is then sutured and closed, followed by removal of the drain after surgery.

### 3.2. Indications

Indications for the axillary approach include the presence of an adenomatous goiter or follicular nodule with a maximum diameter of < 6 cm and a diagnosis of a benign nodule according to fine-needle aspiration cytology [47]. Low-risk papillary microcarcinomas may also be indicated in regions where lobectomy is an accepted practice for these thyroid carcinomas [48]. These tumours must be <15 mm and confined to the thyroid gland, without lymph node metastases, and there must be no evidence of possible local invasion. Patients with Grave's disease may also be indicated for this approach, providing that the gland has a volume <100 ml on preoperative evaluation [48].

### 3.3. Surgical Outcomes

In 2004, Ikeda et al. [47] compared 20 patients who underwent endoscopic total thyroid lobectomy via the axillary approach with 20 patients undergoing MIVAT. The indication for surgery in both sets of patients included follicular nodules <6mm and a diagnosis of benign follicular adenoma on fine-needle aspiration. The surgical invasiveness of each procedure was compared using the operating time, intraoperative blood loss, duration of drainage, length of hospital stay, and degree of pain. The two groups showed no statistical difference in terms of age, gender, and size of thyroid tumour making results comparable. Three months after surgery, the cosmetic results were evaluated and compared.

The operating time for the axillary approach was significantly longer (175 +/- 42 min) than conventional surgery (84 +/- 24 min). None of the patients in the study had any evidence of injury to the RLN or parathyroid glands. Five patients (33%) treated by the axillary approach and 4 patients (27%) who received open surgery complained of neck or anterior chest pain exceeding moderate levels on postoperative day one. One patient, in the axillary approach group, complained of extremely severe chest pain.

All of the axillary approach group were satisfied with the cosmetic results, with 80% being extremely satisfied. Comparatively, 11 out of 15 patients (73%; p < 0.01) in the open surgery group complained about the cosmetic result with two patients reporting extreme dissatisfaction with the operative scar. This study therefore suggests the main advantage of axillary approach is the cosmetic outcome. Other studies have also reported the superior cosmetic benefit of the axillary approach over not only the conventional approach but also other endoscopic procedures [15, 21].

In the largest study to date by Kang et al. in 2009 [22], of 581 patients undergoing transaxillary thyroidectomy, complications rates were comparable to those in the conventional approach; 3.2% of patients had a transient hypocalcaemia, 1.5% of patients had transient hoarseness of voice, and 0.2% of patients had a permanent RLN palsy. Operative time was longer than the conventional approach at 129.4 minutes and 135.5 minutes for benign and malignant tumours, respectively.

A potential benefit of the lateral axillary approach, over MIVAT, is that larger tumours or nodules can be extracted through the 30mm axillary incision, meaning more patients are indicated for this approach [46, 49]. The lateral view of the thyroid, during the axillary approach, may have some advantages such as easy identification of the ipsilateral RLN and parathyroid glands [49]. Successful central lymph node dissection has been reported in a series of 37 patients [50]. However, this approach does have some drawbacks. Ikeda et al. [47] described difficulties in dissecting the contralateral lobe of the thyroid gland and found a considerably increased operating time. They reported that the limited unilateral view and the restricted operative field mean that performing a total thyroidectomy via the axillary approach is challenging because visualisation of the contralateral RLN is poor and the operating time is unfeasibly long.

Technical difficulties are another potential disadvantage of the axillary approach. The three trocars are inserted very close together, there is only a narrow operating space, and interference of the surgical instruments occurs frequently [16]. This so-called “sword fighting” and the fact that all manoeuvres have to be performed from one direction have been proposed as potential reasons for the longer operative time seen in such procedures, even in the hands of the most skilled surgeons [15].

To date, several factors have limited widespread application of the axillary approach including insufficient working space, need for special equipment, and the need for skilled surgical training; the axillary approach gives a lateral view of the thyroid that will be atypical to most surgeons [10]. Alongside this, the axillary approach operating time exceeds not only conventional thyroid surgery but also other ET procedures [51, 52]. The question remains whether the cost of surgical instruments, longer operating time, and cost of surgical training truly outweigh the superior cosmetic result.

## 4. Robotic Transaxillary Thyroidectomy

The transaxillary approach was first pioneered by a South Korean team led by Professor Chung in 2007 [29], initially being performed via an axillary and separate anterior chest incision. The approach has since been modified to a single axillary incision. The largest study to date was reported by Ban et al. [24], of 3000 patients undergoing robotic axillary thyroid surgery by Professor Chung and team. The patient selection was mainly low BMI (average 22kg/m^2^) on relatively small thyroid nodules (average 0.66cm); the average time for total thyroidectomy was 141 minutes with complications rates reported as similar to conventional thyroidectomy.

The use of robotics can overcome some of the limitations of extracervical approaches; advantages include a three-dimensional view of the operating field, a greater degree of movement from the use of ‘wristed' instruments, and the elimination of hand tremors [24, 26–28]. Advantages of this approach include ease of detecting the RLN and parathyroid glands [29], easier manipulation of the upper and lower poles of the thyroid, and ability to perform total thyroidectomy with central and lateral neck dissections for advanced cancer. The safety and feasibility of the approach are well described with rates of complications (blood loss, hypocalcaemia, RLN injury) seen comparable to that of conventional approaches [53].

The main disadvantages of the robotic axillary approach are possible risks of anterior chest paraesthesia and brachial plexus injury and potential complications of tracheal and oesophageal injury. Anterior chest paraesthesia is an unavoidable complication of this approach due to damage to sensory nerves of the cervical plexus chain that are encountered during creation of subplatysmal working space. For the majority of cases, the paraesthesia is temporary, though permanent cases have been reported. In addition, due to the ipsilateral arm position, there is the risk of brachial plexus injury. Despite a low incident (0.2%) and transient nature being reported in the literature thus far, it is still significantly worrying complications [54, 55]. The risk of brachial plexus injury has since been reduced by placing the arm in a flexed 90-degree position (extended salute position), which reduces the traction on the nerve [56, 57]. Rare complications of tracheal and oesophageal injuries have been reported; however, the incidence is no higher than the conventional open approach [58].

A meta-analysis and systematic review published by Jackson et al. in 2014 [59] summarized a total of nine studies comparing the robotic transaxillary thyroidectomy to endoscopic cervical thyroidectomy and conventional thyroidectomy. The analysis included a total of 2,881 patients of whom 1,122 underwent robotic thyroidectomy. Operative time was longer for robotic thyroidectomy than the conventional approach, by 42 minutes, but no significant difference was found between robotics and endoscopic thyroidectomy. Postoperative complication rates were comparative between all groups. Length of stay was significantly increased in open thyroidectomy; the length of stay was comparable between endoscopic and robotic approaches. Cosmetic satisfaction was also found to be considerably higher in robotic transaxillary thyroidectomy patients compared with open thyroidectomy. The outcomes of the study concluded that robotic axillary approach thyroidectomy is a safe and feasible alternative to endoscopic and open thyroidectomy, with comparative postoperative complications, shorter hospitalization, and higher patient satisfaction [57]. Studies have analysed the learning curve associated with robotic transaxillary thyroidectomy. In the largest study by Kandil et al. [59], a decrease in total operative time was found after 45 cases.

## 5. Endoscopic Axillo-Breast (Hybrid) Approach

### 5.1. Surgical Pathway

In 2003, Shimazu et al. [15] presented results of a technique termed the axillary-bilateral breast approach (ABBA). The ABBA is a modified version of the anterior/breast approach whereby the parasternal incision is converted to an axillary incision on the pathological side.

Under general anaesthesia, the patient is placed supine and both arms abducted. A 2.5 cm circumareolar incision is made on the ipsilateral side. Blunt dissection with a balloon dissector is then used to create the subcutaneous and subplatysmal working space. A 10 mm trocar is then inserted and the working space is maintained with low-pressure (4-6 mmHg) CO_2_ gas insufflation. Two further 10 mm trocars are then inserted via an ipsilateral axilla and contralateral circumareolar incision. The rest of the procedure follows a similar approach to the anterior chest/breast approach; however, the additional axillary port provides a wider triangulation of instruments to facilitate dissection and mobilization of the thyroid gland. The resected thyroid gland is eventually removed via the circumareolar wound on the ipsilateral side.

### 5.2. Indications

Since its introduction, indications for the axillo-breast have included low-risk (nonmetastatic) thyroid carcinomas not larger than 1cm, follicular neoplasms less than 3cm, and benign thyroid masses. The upper limit of thyroid volume for inclusion was defined as 40ml in one study [60]; however, this was poorly defined in other studies [15, 16].

### 5.3. Surgical Outcomes

One of the reasons for this modification was that the parasternal scar often became hypertrophic following the anterior/breast procedure (this is not the case with the upper areolar incisions) [15]. Shimazu et al. [15] presented the results of the ABBA technique in 12 patients and compared this to 4 patients who underwent thyroidectomy via the anterior/breast approach. All patients were female with an average age of 35 years (range 15-52); all patients had a preoperative diagnosis of follicular carcinoma. All procedures were successful; neither group reported any intraoperative complications or postoperative complications. The authors reported better cosmesis in the ABBA group, due to the axillary scar being completely covered by the arm; in contrast, two patients (50%) in the anterior/breast approach group showed hypertrophic parasternal site. Due to the disparity in the size of the two patient groups studied, the reported superior cosmesis must be viewed with caution.

Shimazu [15] reported that the axillary incision not only improved cosmesis but also allowed a better view and reduced interference from surgical instruments. The multiangle approach provided easier handling of surgical instruments, avoiding the “sword fighting” in the axillary approach. This was reflected in a significantly shorter operation time in the ABBA group (188 min vs. 270 min, p < 0.01). Further to this, Barlehner et al. [60] published results of 13 successful thyroidectomy procedures via the ABBA in a 2008 study. Six of these procedures where bilateral thyroidectomies; the ability to remove larger specimens and operate bilaterally could mean wider application of this approach as more thyroid diseases are likely to be indicated.

## 6. Endoscopic Bilateral Axillo-Breast Approach (BABA)

Choe et al. [16], in 2007, modified the ABBA by developing the bilateral axillo-breast approach (BABA) to obtain optimal visualization for total thyroidectomy by adding a contralateral axillary port. A total of 102 patients were treated by this method and results were compared to those of 25 patients undergoing the ABBA procedure. They reported excellent cosmetic results; there were no hypertrophic scars in any of the 102 patients, and 76.5% (78 of 102) reported the cosmetic result as excellent. A further study by Choi et al. [61] was published from a larger series of 512 patients. This reported a low rate of permanent complications such as permanent RLN palsy (1.7%) and hypocalcaemia (4.2%). The rate of transient hypercalcaemia was 31.1% and rate of transient RLN palsy was 20.1%; the facial nerve was also susceptible to traction injury during the operation.

Several different authors have reported better exposure using this technique with the view and orientation similar to that observed in conventional thyroidectomy. Further to this, the symmetrical approach reduces interference between instruments during the procedure [17, 61]; central lymph node dissection has since been performed using the BABA approach [62]. One potential issue with the techniques, however, is the scarring of the breast. Despite the upper areolar incisions rarely forming hypertrophic scars in the ABBA and BABA [15, 35], some patients, especially young female patients, are reluctant to have scars on their breasts [63]. In addition to this, both operations are technically challenging and even though they avoid the periareolar dissection, these techniques have not proved as popular as the transaxillary approach.

## 7. Endoscopic Postauricular and Axillary Approach

### 7.1. Surgical Pathway

In 2009, Lee et al. [17] presented the first results of a postauricular approach in 10 patients. This technique utilizes two axillary and two postauricular ports thus avoiding breast incisions. A 12 mm axillary incision is made at the lesion side and the subcutaneous space is made via blunt dissection with a vascular tunneler. Following this, a 12 mm trocar is inserted into the axillary incision and low-pressure CO_2_ (5-6 mmHg) is used for insufflation. A contralateral axillary incision is made with insertion of a 5 mm trocar. Bilateral postauricular incisions are completed with insertion of two 5mm trocars.

A midline incision is performed between the strap muscles from the suprasternal notch to the thyroid cartilage. The bilateral strap muscles are then retracted laterally by right-angled graspers through the postauricular ports. Ultrasonic shears are used to divide the midline isthmus and then used to coagulate the superior vessels of the thyroid gland. Identification and protection of the RLN and parathyroid glands preclude resection of the thyroid. The resected thyroid is excised in a similar fashion to the conventional anterior approach and removed via the larger 12 mm axillary port. A contralateral thyroid lobectomy is then performed via the same technique. The midline incision is repaired with endosutures, and a suction drain is left in place, with final closure of the initial incisions.

### 7.2. Indications

Indications for the postauricular and axillary approach include benign thyroid masses <4 cm in its largest diameter, low risk papillary thyroid carcinoma <1 cm, follicular neoplasm <3 cm, and parathyroid adenoma localized preoperatively [17].

### 7.3. Surgical Outcomes

The initial study by Lee et al. [17] reported a small series of 10 patients using the axillary and postauricular approach with a total of 7 bilateral thyroid resections being undertaken. All patients were female; seven patients had a papillary thyroid microcarcinoma, one with parathyroid adenoma, one with microinvasive follicular thyroid carcinoma, and one with an adenomatous goiter. The authors reported that the estimated blood loss from the surgery was minimal; no cases of conversion to open surgery were described. The average operative time was 210.0 ± 43.7 min; this was longer than the aforementioned axillary and anterior/breast approaches. The authors noted, however, that this was a shorter operative time than the BABA approach first endoscopic cases and suggested that operating times were likely to become shorter as techniques and instruments evolved. Vocal cord paralysis was monitored pre- and postoperatively with 3 patients observed to have a transient hoarseness of voice which resolved by 1 month in all cases; however, the use of intraoperative neuromonitoring was not described in the operative technique which may have contributed to a higher rate of transient RLN palsy. Transient hyperaesthesia was noted in the dissected area in the distribution of the great auricular nerve, which was managed with pain control and reported to be normal within several months after surgery. The average time to discharge was 3.4 days ± 1.07. The study stated excellent cosmetic results due to the hidden location of incisional scars; however, no patient reported outcomes were documented. One of the concerns with dissecting the postauricular area is the close proximity of the facial nerve: although no permanent cases of facial nerve injury were reported in Lee's study, patients did complain of sensory loss in the dissected area; the authors claim this returned to normal within several months but did not quantify an exact time frame.

The approach by Lee et al. [17] was developed with the aim of advancing the BABA approach. The heralded advantages of the BABA approach were maintained with a bilateral and symmetrical view of important anatomical structures combined with a medial approach which is similar to that permitted by open thyroid surgery. The development of this new technique enabled avoidance of scaring around the breast/areolar region with the added advantage that the postauricular dissection offered a route of dissection which is familiar to head and neck surgeons [64].

### 7.4. Robotic Retroauricular ‘Facelift' Approach

In 2011, Terris et al. [65] described a robotic retroauricular (facelift) approach in an initial series of 14 patients, a development of the postauricular approach described by Lee et al. in 2009 [17]. The aim was to help overcome the limitations that were reported with the robotic transaxillary approach [65]. A larger series by Kanhil et al. in 2012 [59] reported 91 patients undergoing robotic retroauricular (facelift) approach. The mean operative time was 108 mins and 118 mins for hemithyroidectomy and total thyroidectomy, respectively. There were two cases of conversion to open thyroidectomy. No instances of permanent vocal cord paralysis were reported.

Advantages over the transaxillary approach include significantly reduced field of dissection. Singer et al. [66] reported a reduced dissection area of 38%. Further to this, retroauricular surgery is considered a more surgically familiar approach which is widely used in parotidectomy and submandibular gland incision [67–69]. The facelift approach may be more appropriate for patients with higher BMI with authors commenting that the technique is easier to perform on obese patients than the transaxillary approach [65]. There is no risk of brachial plexus injury and no concern of paraesthesia of the anterior chest unlike the transaxillary approach [64, 70]. There are, however, some disadvantages that are inherent to this approach; transient hyperaesthesia in the distribution of the greater auricular nerve is universal. Though transient, patients need to be counseled appropriately as this does not occur through the conventional open approach [65, 71]. Another limitation is that due to the vector of the approach bilateral incisions are required to perform total thyroidectomy compared to the singular incision needed for total thyroidectomy in the axillary approach [72].

## 8. Endoscopic Transoral Approach

### 8.1. Surgical Pathway

In 2010, Wilhelm [73] was the first surgeon to perform transoral thyroid surgery in clinical practice, with removal of a right-sided thyroid tumour in a 52-year-old male. The aim of this approach was to develop a completely scarless technique and reduce the extensive neck dissection seen in previous extracervical approaches. In the technique described by Wilhelm et al. [73], a midline sublingual incision is made and a 5 mm trocar is then placed into the subplatysmal layer, through the floor of the mouth. CO_2_ insufflation at 6 mmHg is used to create the operating space. A second incision is then made in the vestibular mucosa allowing passage of a second trocar. Following careful dissection of the surgical field, a third trocar is placed in the vestibule of the mouth on the contralateral side. The thyroid capsule is exposed through a midline incision through the linea alba and strap muscles dissected away to expose the right upper lobe of the thyroid gland. The isthmus of the thyroid is divided using a harmonic scalpel and upper pole vessels divided. The RLN is then visualized and intraoperative neuromonitoring is used to assess nerve function. Following release of the lower pole of thyroid, the tumour is removed via the medial trocar incision, and wounds are then closed with absorbable sutures.

### 8.2. Indications

Indications for the transoral approach include patients with follicular tumour, symptomatic large goiter, Grave's disease, and papillary microcarcinoma without evident lymph node metastases [74]. A 6 cm maximum diameter nodule size is commonly utilized [75, 76].

### 8.3. Surgical Outcomes

The patient in Wilhelm's [73] initial study showed no complications except for minimal neck swelling and haematoma. Following this, the same team conducted a further study of eight patients undergoing total and subtotal thyroidectomies [77]. During this study, one case of permanent RLN palsy was observed. There were also six cases of transient mental nerve palsy reported and one case of RLN palsy observed which resolved on follow-up. The average operating time was reported at 239 mins; three cases were converted to open thyroid surgery due to large tumour size.

Following this first clinical application of transoral surgery, several variations of surgical technique have been developed. A study of 8 patients using a new gasless transoral video-assisted neck surgery (TOVANS) approach was published by Nakajo et al. [74]. No cases of hypoparathyroidism, wound infection, or mental nerve palsy were reported. One case of temporary recurrent laryngeal nerve palsy was reported. Richmon et al., in 2011, first described a transoral vestibular approach, moving all incisions away from the floor of the mouth, in cadaveric studies [78]. Park et al. [79] adopted the modified trivestibular approach in 2014, with the first clinical application of this technique on a 30-year-old female, without any complications in 2016, by the same team. Park et al. [80] have recently published a report of 18 individuals undergoing the trivestibular approach where tumours as large as 7.5cm were successfully removed transorally. No cases of RLN or mental nerve palsy were reported and there were no cases of surgical site infections. Postoperative complications included one case of transient hypocalcaemia and two cases of seroma.

The largest clinical cases series, to date, was published by Anuowng in 2018 [81], involving a series of 425 patients undergoing transoral endoscopic thyroidectomy via the vestibular approach. The operative time was 100 min (SD 39.7). The postoperative complication rate was also reduced compared to initial studies; forty-six (10.9%) cases of temporary hypoparathyroidism were observed, twenty-five (5.9%) patients had temporary RLN palsies and one patient was observed to have haematoma formation. Three patients (0.7%) had transient mental nerve injury. No cases of permanent recurrent laryngeal nerve palsy or permanent hypoparathyroidism were reported.

Since the first description of the transoral approach, there has been significant interest in the technique, particularly in Eastern Asia, due to the potential to allow surgeries without skin incisions [82]. Whilst several different approaches have been tried, the transoral vestibular approach proves to be the most popular. Another benefit is the shorter dissection tunnel and minimally invasive approach to the more established extracervical approaches; techniques such as the axillary approach require extensive tissue dissection to expose the thyroid gland and therefore some authors argue they are not truly minimally invasive [83]. Complete endoscopic radical lymphadenectomy for papillary thyroid cancer has also been reported using the transoral technique [74], giving the potential for wider indications of this technique.

There are, however, disadvantages to this approach. Firstly, the craniocaudal approach is foreign to the conventional approach to open thyroidectomy known to head and neck surgeons. It therefore requires a comprehensive knowledge of anatomical structures and may prove to be technically challenging. Kahramangil et al. [83] state that, despite the direct access to the neck, the dissection of the lateral borders of the thyroid lobes was more difficult in the transoral approach compared to the axillary approach.

Another potential drawback is the potential for communication of oral infections into the anterior neck area; however, in studies to date, surgical site infections have not appeared to be problematic [74, 77, 80, 81]. Mental nerve palsies are also a complication not seen in axillary and breast approaches and have been reported in two case series [75, 77]. A modification of technique by Anuwong [81] in 2016 repositioned the lateral trocar towards the edge of lip (thus reducing tension on the mental nerve) and reported no cases of permanent RLN palsy in the aforementioned case series of 60 patients. This modification has been widely accepted and has greatly reduced chances of mental nerve palsy. In a systematic review by Shan et al. [84], the overall incidence of the main two complications transient hypoparathyroidism (7.4% across studies) and temporary/permanent RLN palsy (4.3% across studies) were not shown to be higher than incidences reported in conventional open thyroidectomy studies [85]. To date, the largest benign tumour removed was 7.5cm by Park et al. in their 2017 study; however, no malignant tumour larger than 2.5cm has been removed transorally [80]. This potentially limits the widespread application of this approach.

To conclude, the transoral approach has been shown to be safe and feasible in recent studies and postoperative complications are at an acceptable limit compared to initial studies. The undoubted superior cosmetic outcomes of an externally scarless approach may lead to further implementation in the future; however, more clinical studies are needed to fully examine its feasibility and potential limitations.

### 8.4. Robotic Transoral Thyroidectomy

Transoral thyroidectomy is the latest concept that has attracted significant interest. Following animal and cadaveric studies, in 2015, Lee et al. [86] reported the first experience of performing transoral robotic thyroidectomy in 4 patients. Despite successful completion of operations, three of the four patients suffered temporary paraesthesia in the distribution of the mental nerve. Concern over mental nerve injury in this study initially halted further widespread uptake of this technique until Anuwong [81] pioneered a modification of the transoral endoscopic technique in 2016. Anuwong modified the approach by positioning of the lateral trocars towards the free edge of the lip to prevent excessive tension of the mental nerves. This modification helped solve the problem of mental nerve paraesthesia enticing further studies into the potential of robotics via the transoral approach. A limited number of studies are available thus far; a study undertaken by Richmon et al. in 2017 [87] reported a small series of 17 patients. The mean operating time was 254 mins, with one conversion to open thyroidectomy. The average nodule size was reported as 1.2 cm. Complications included hyperaesthesia of the lower lip (n=3), lip weakness (n=1), a small lateral lip tear which healed (n=1), bruising over the zygomatic regions (n=1), and perforation of the chin skin (n=1).

The obvious advantage of the transoral approach is a complete avoidance of skin incision, with invisible intraoral scars. The approach provides this without significantly increasing the amount of required dissection whilst accessing the thyroid from a natural orifice [82, 88]. The transoral approach provides equal access to both sides of the neck allowing excellent exposure to bilateral thyroid lobes for total thyroidectomy compared to other approaches.

One of the main disadvantages of this technique is the potential introduction of oral infectious agents into the neck requiring the need for postoperative antibiotics. Postoperative length of stay is considered to be longer than other approaches with patients being discharged 1-3 days after procedure [88]. This is due to dietary restriction (liquid then soft diet) in the first 24 hours and required drain removal three days postoperatively [89, 90]. One of the biggest weaknesses is difficulty in dissection of the lateral borders of thyroid which is technically challenging [88].

## 9. Discussion of Endoscopic Approaches

In the last two decades, extracervical approaches have gained considerable interest with numerous aforementioned techniques being described. As discussed, each technique has its own individual advantages and disadvantages. Extracervical approaches have continued to evolve with an increasing body of research [43]. For example, primary indications initially included benign thyroid nodules with a maximum diameter of 6cm and maximum thyroid gland volume of 60ml [16, 21, 46, 49]. As operative experience has increased, larger tumours (up to 10cm in diameter), goiters, and low-grade malignancies have all successfully been attempted [13, 91, 92]. Common contraindications include previous surgery or irradiation and invasive malignant tumours [42]. Surgery in obese patients is technically more challenging with some authors advocating avoidance in high body mass index patients [93].

Feasibility of bilateral thyroid dissection differs from each approach. Lateral approaches such as the axillary approach have poor visualization of the contralateral thyroid lobe and therefore poor identification of the contralateral RLN which limits bilateral thyroidectomy being performed in such approaches [61]. More recent approaches including the BABA and transoral approaches have more symmetrical approaches allowing for bilateral thyroid dissection and potentially larger nodules [91].

Low-risk thyroid carcinoma inclusion in extracervical approaches indications is becoming more commonplace in recent studies. Oncological risks include seeding and local recurrence and therefore thorough evaluation of these risks is imperative. The ability to perform surgery for differentiated thyroid carcinoma is dependent on successful central lymph node dissection. The central neck lymph nodes are defined by the hyoid bone superiorly, the carotid sheath laterally, and the innominate artery inferiorly [94]. This is a high risk area due to the presence of the RLN and parathyroid glands. Successful central neck lymph node dissection has been reported in extracervical approaches [22, 95] with reported recurrence rates comparable to conventional approaches. Larger scale evaluation of the feasibility and safety of central lymph node dissection is however needed. Thyroid cancer can also metastasise to the lateral lymph nodes. Suspicious lymph nodes in the lateral compartment should be biopsied preoperatively by fine-needle aspiration [94]. Whilst studies have reported successful cases of lateral lymph node dissection in endoscopic surgery [96], at present, lateral lymph node dissection is not recommended in endoscopic approaches due to inadequate exposure [38]. Thus, prior identification of lateral lymph node involvement limits the indications of extracervical approaches with requirement of a modified radical lymphadenectomy in these patients [97].

Another potential disadvantage is the longer operative time and considerable learning curve associated with extracervical approaches. All techniques require a larger dissection area and resultant longer surgical approach leading to increased operative time [98]. With all reported techniques included in this review, increased experience and improved surgical techniques will reduce operative time closer and closer to that of conventional techniques. The operative time, however, will invariably be longer for extracervical approaches and the benefit of improved cosmesis must be balanced against the financial implications of longer operations.

Operative complications in extracervical approaches are similar to those found in conventional thyroidectomy with added unique complications of subcutaneous emphysema, chest wall paraesthesia, and seroma (14,17 from Johri 2018). Initial studies reported higher rates of complications [17, 39–43] which have reduced to rates comparable to conventional techniques with improvement of surgical skills and instruments [98]. Perhaps the most important complications of thyroid surgery are parathyroid gland and RLN injury. Geng-Zhen et al. [98], in 2011, found no significant difference in rates of hypocalcaemia and RLN injury rates between cervical and extracervical approaches in a systematic review. The authors suggested that a magnified operative view with endoscopic surgery allows easier identification of the RLN and parathyroid glands reducing injury frequency. Application of RLN monitoring to extracervical techniques is becoming more commonplace (35 from Geng-Zhen) and has been shown to reduce rates of RLN injury in some small sample studies [99, 100].

Yeung et al. [63] have highlighted the impact of culture on cosmetic outcome. Western and Asian young female patients may still find scarring of the breast area unacceptable; thus, the transoral approach may be of significant value in avoiding scarring, especially in regions or countries where the desire for cosmesis is high. The impact of social stigma towards visible scars is a highly relevant issue for extracervical approaches. Foley et al. [101], for example, discuss the significant social stigma in Korea and other far eastern countries associated with visible scars in the neck. In contrast, an American study by Linos et al. [102], in 2013, on 691 patients on scar perceptions after thyroid and parathyroid surgery showed high levels of patient satisfaction. The study compared direct endoscopic surgery with conventional open surgery. Patient satisfaction with scarring was similar regardless of approach. Most patients (81.2%) reported that they would not have preferred a transaxillary procedure over the procedure they received. The authors conclude that new surgical approaches aimed at maximizing cosmesis while minimizing scar size should be evaluated for cost-effectiveness and clinical outcomes, as well as patient satisfaction, before becoming the standard of care. It is therefore likely that cultural perceptions will influence the extent of adoption of extracervical approaches to thyroid surgery.

At present, uptake of extracervical approaches is limited, with variation around the world in which technique is most commonly utilized. In Asia, for example, the transaxillary approach is still being used whilst it is used very sparingly in the United States. By far, the fastest growing and most commonly utilized approach is the transoral endoscopic approach.

## 10. Discussion of Robotic Approaches

When considering the advantages of robotic approaches, it is first important to consider the benefit the robotic system gives. The commonest robotic system used, the da Vinci system (Intuitive Surgical, USA), offers a 3-dimensional 10-time magnified view, with wristed robotic arms with 7 degrees of freedom and tremor elimination which may enhance the safety and precision of the procedure [103]. Superior visualization and precise tissue manipulation have been reported with the da Vinci system during robotic transaxillary thyroidectomy [57]. There are, however, disadvantages cited in the literature that warrant further discussion.

Two of the most commonly discussed potential drawbacks of robotic thyroid surgery are the longer operative times required for procedures and the steep learning curve required. Extracervical robotic thyroidectomy, even in the hands of the most skilled surgeon, adds around 45 minutes to operative times [104]. Whilst this has not been shown to bear any impact on patient outcomes, quality of life. or length of hospital stay, longer operative times increase associated facility and staffing fees adding to the already high costs of robotic surgeries. A steep learning curve has been demonstrated for robotic transaxillary thyroidectomy; with reduction in operating time after 40 to 45 cases [59]. The number of cases needed to achieve this plateau may limit its widespread application to highly specialized centres and a small number of surgeons. Cabot et al. [105] determined that in order for robotic thyroidectomy via the transaxillary approach to be cost equivalent to the conventional approach the operative time would have to be reduced by nearly half compared to current operative times.

Early adoption of the technique from Eastern procedures failed to account for important anthropometric differences between the two populations, namely, the larger BMI of individuals in Western societies and larger thyroid nodules. The larger thyroid nodules are accounted for by the presence of a national thyroid cancer screening programme in South Korea [56] giving earlier detection rates. The increased body habitus has proved to be problematic; a study by Kandil et al. [106] found a significantly increased operative time in robotic transaxillary thyroidectomy patients who had a BMI greater than 30, raising concerns regarding the applicableness to robotic thyroid surgery in obese patients. Several studies since, however, have demonstrated that in experienced hands operation times can be reduced in obese patients undergoing robotic transaxillary thyroidectomy [59, 106, 107].

The largest barrier to robotic thyroidectomy, to date, is prohibitive cost. Cabot et al. [105] reported the costs of the robotic approach (transaxillary) are significantly higher than that of conventional open thyroidectomy primarily due to related equipment costs and longer operative times. The significant cost of the robotic system may be reduced in future. To date, all remote-access thyroid surgery has been performed by the da Vinci system (Intuitive Surgical, USA) [108] and the costs of robotic surgery are likely to be driven down in future with increased competition from medical device companies that are entering the surgical robotics market [109]. In the context of growing health expenses throughout the Western world, cost efficiency is a major concern for healthcare providers; currently, the cost inefficiency of robotics is likely to limit its widespread application.

## 11. Conclusion

Extracervical approaches to thyroidectomy are yet to be widely employed in clinical practice worldwide; however, the drive to improve cosmesis is still important in some patients and particularly in cultures where visible scarring is socially stigmatised. The cosmetic superiority in avoiding visible scarring must be balanced against increased expense, operative time, and the need for surgical training. Extracervical approaches require substantial experience in endoscopic surgery to obtain satisfactory results; however, it is a valid and feasible option for selected patients whose desire for cosmesis is high. The role of robotic systems in extracervical thyroidectomy is still unclear; further studies are needed to assess whether robotics adds a considerable enough benefit to patient outcomes to negate the higher expenditure costs that come with robotic surgery. For now, these techniques are limited to high-volume centres, with adequate experience to achieve the cosmetic advantages that are heralded by such approaches.
